# The significant association of *Taq*1A genotypes in *DRD2/ANKK1* with smoking cessation in a large-scale meta-analysis of Caucasian populations

**DOI:** 10.1038/tp.2015.176

**Published:** 2015-12-01

**Authors:** Y Ma, M Wang, W Yuan, K Su, M D Li

**Affiliations:** 1 State Key Laboratory for Diagnosis and Treatment of Infectious Diseases, The First Affiliated Hospital, Collaborative Innovation Center for Diagnosis and Treatment of Infectious Diseases, Zhejiang University School of Medicine, Hangzhou, China; 2 Air Center for Air Pollution and Health, Zhejiang University, Hangzhou, China; 3 Department of Psychiatry and Neurobehavioral Sciences, University of Virginia, Charlottesville, VA, USA

## Abstract

Although a number of studies have analyzed the relation between the *DRD2*/*ANKK1* gene *Taq*1A polymorphism and smoking cessation, the results remain controversial. The primary objective of the present study was to determine whether this variant indeed has any effect on smoking cessation. The A1-dominant model that considers A1/* (*=A1 or A2) and A2/A2 as two genotypes and compares their frequencies in current and former smokers was applied. A total of 22 studies with 11 075 subjects were included in the meta-analyses. Considering the potential influence of between-study heterogeneity, we conducted stratified meta-analyses with the Comprehensive Meta-Analysis statistical software (version 2.0). Results based on either cross-sectional or longitudinal studies consistently showed a statistically significant association between *Taq*1A A1/* genotypes and smoking cessation. Further, a more significant association of the variant with smoking cessation was detected when both types of studies were combined. However, there was marginal evidence of heterogeneity among studies (*I*^2^=33.9% *P*=0.06). By excluding other ethnicities and subjects with cancer, the meta-analysis on the basis of 9487 Caucasians demonstrated that *Taq*1A A1/* genotypes indeed were significantly associated with smoking cessation under both the fixed- and random-effects models (pooled OR 1.22; 95% CI 1.11–1.34; *P*=3.9 × 10^−5^ for both models). No evidence of between-study heterogeneity or publication bias was observed. Thus, we conclude that the polymorphism of *Taq*1A has an important role in the process of abstaining from smoking, and smokers carrying A2/A2 genotype have a higher likelihood of smoking cessation than those who carry A1/A1 or A1/A2.

## Introduction

Cigarette smoking is the most common preventable cause of many diseases that contribute to about 6 million deaths worldwide each year.^1^ Twin and family studies indicate that smoking behaviors are influenced by both genetic and environmental factors.^2, 3, 4^ There exists a considerable interest in identifying genetic factors encouraging smoking cessation, of which the heritability is estimated to be ~50%.^5, 6^

The dopaminergic reward system has a crucial role in the etiology of smoking initiation and the subsequent development of nicotine dependence. A great number of studies have focused on determining whether variants in genes with a dopaminergic function could contribute to the heritable variation of smoking behaviors. This reward system consists of three parts: dopamine receptors, dopamine transporters and enzyme targets, which jointly modulate the concentrations of dopamine in synaptic clefts. Because of increasing synaptic dopamine concentrations to a higher level than those stimulated by natural rewards such as sexual intercourse and food, nicotine from cigarette smoke is considered a partial moderating factor of smoking maintenance, and variants in those genes linked with increasing the concentrations of synaptic dopamine appear to be more likely to be involved in smoking cessation.

Of these genes, *DRD2*, located on chromosome 11q22–q23, has received much attention.^7, 8, 9^ Many studies^10, 11, 12, 13, 14, 15, 16^ have reported that a growing number of variants in *DRD2* are significantly associated with smoking behaviors. In particular, the *Taq*1A polymorphism (dbSNP rs1800497), located ~9.5 kb downstream from *DRD2* and in exon 8 of *ANKK1*, has been investigated extensively. Since Noble *et al.*^17^ first showed that there was a significantly higher prevalence of *Taq*1A A1 allele among current smokers (*P*=0.024) and former smokers (*P*=0.044) than among nonsmokers in the Caucasian population, many genetic association studies^15, 18, 19, 20, 21, 22, 23, 24, 25, 26, 27^ were conducted to elucidate the association between the polymorphism of *Taq*1A and smoking-related phenotypes, including cessation. However, the results remain ambiguous. For example, two meta-analyses^28, 29^ showed no evidence of association between *Taq*1A polymorphism and smoking behaviors, whereas other two meta-analyses^30, 31^ showed significant associations of *Taq*1A polymorphism with smoking behaviors. In addition, there exists heterogeneity among the findings of reported studies.

Both *in vivo* and *in vitro* studies^32, 33, 34, 35^ have documented that the minor A1 allele (or T allele) of the *Taq*1A polymorphism is correlated with regulating the density and availability of dopamine D2 receptor, indicating that the variant potentially involves tailoring of the concentrations of synaptic dopamine. Therefore, it is rational to assume that the *Taq*1A polymorphism has an important part in regulating smoking cessation. A growing body of pharmacogenetic evidence^36, 37, 38, 39^ has suggested an association of *Taq*1A polymorphism with the pharmacotherapy of smoking cessation, although a few longitudinal treatment studies^40, 41^ have failed to reveal such an association. Many moderating factors could be causing these inconsistent results, such as gene × sex^36, 42^ and *DRD2* × *SLC6A3* interactions.^38, 43^

To date, there have been three meta-analyses^28, 31, 44^ regarding the association of *Taq*1A polymorphism with smoking cessation. On the basis of five cross-sectional studies with relatively small samples (*N*=1750 in total), the first reported meta-analysis by Munafo *et al.*^28^ found no evidence of an association between the variant and smoking cessation. Then Ohmoto *et al.*^31^ performed an independent meta-analysis by pooling six cross-sectional studies and one longitudinal treatment study, of which only the placebo arm was employed (*N*=4249). The authors demonstrated that smokers with one or more A1 alleles have more difficulty of quitting smoking than do those carrying homozygous A2 alleles in both the overall population (pooled odds ratio (OR) 1.19; 95% confidence interval (CI) 1.05–1.35; *P*=0.008) and the Caucasian population (pooled OR 1.27; 95% CI 1.10–1.45; *P*=0.001). Very recently, a meta-analysis by Choi and Shin^44^ integrating nine longitudinal treatment studies of which the placebo arms were excluded (*N*=2851), showed that there was no significant association between *DRD2 Taq*1A polymorphism and smoking cessation outcomes. Because no meta-analysis was conducted combining the effects from both longitudinal treatment and cross-sectional studies, and several related studies were recently reported, it is important to conduct an updated meta-analysis for the effect of *Taq*1A polymorphism on smoking cessation. Thus, we performed a stratified meta-analysis by considering the influence of the underlying sources of heterogeneity such as method variation, ancestral variation and sampling variation.

## Materials and methods

### Search strategy and inclusion criteria

We searched the reported studies on the association of *DRD2*/*ANKK1 Taq*1A polymorphism with smoking by interrogating the electronic database of PubMed. From the first date available in the database up to 17 May 2015, the search was performed using the following key terms: ‘dopamine D2 receptor,' ‘*DRD2*,' ‘smoking,' ‘nicotine,' ‘cigarette' and ‘tobacco.' Electronic abstracts were examined for potentially relevant articles in accordance with the standard inclusion and exclusion criteria suggested by Moher *et al.*^45^ Duplications were discarded. Once studies met the criteria for selection, the references were hand-checked for underlying additional studies. Articles that reported previously published data were excluded.

### Data extraction

After a systematic review according to the Quality of Reporting of meta-analysis and PRISMA guidelines (Supplementary Figure S1), 29 studies were examined more closely for possible inclusion. After excluding the repeated data set, such as data from the study reported by Yudkin *et al.*,^36^ which were the same data used by Johnstone *et al.*,^37^ 23 eligible studies, consisting of nine cross-sectional surveys,^15, 17, 19, 20, 21, 22, 23, 46, 47^ one longitudinal no-treatment study^48^ and 13 longitudinal treatment studies^36, 38, 39, 40, 41, 43, 49, 50, 51, 52, 53, 54, 55^ satisfied the inclusion criteria for this meta-analysis. As described in many previous studies,^28, 31, 56, 57^ we used a comparison of current smokers (non-abstinent group) with former smokers (abstinent group) to define the phenotype of smoking cessation for all acceptable studies. For each study, the following data were extracted by two authors (YM and MW) using standardized forms: authors and year of publication, language (English or other), country of origin, type of study, ancestry of sample, sample size, sex ratio, statement of Hardy–Weinberg equilibrium, diagnostic criteria, classification of smoking status and the number of participants having different smoking statuses categorized by the *Taq*1A genotype (Table 1; Supplementary Table S1).

### Genotype and phenotype classification

In the accepted studies, the genetic model was mainly assumed to be an A1-dominant model saying that subjects carrying one or more A1 alleles were less likely to have achieved smoking cessation than were those who carry no such allele. We thus applied the A1-dominant model where the comparison is A1/* genotypes, including A1/A1 and A1/A2 genotypes, compared with the A2/A2 genotype. In the cross-sectional studies, the classification of smoking status was based on self-reported questionnaire responses, whereas except for a longitudinal treatment study by Swan *et al.*^43^ classifying smoking by self-report, in other longitudinal studies, both self-reported responses and biochemical verification, including the measurements of expiratory carbon monoxide or salivary cotinine concentration, were applied. Of note, only smokers who volunteer to stop smoking were recruited in the longitudinal treatment studies.

Because smoking cessation followed by relapses is a dynamic process, the cessation rate in the treatment study declines as the follow-up time increases after the pharmacotherapy. The included treatment studies consisted of a variety of time frames for smoking abstinence assessment, which may be one of the underlying limitations for this meta-analysis. Considering that former smokers were generally classified as those who had stopped smoking at least 12 months before the survey in cross-sectional studies, in order to harmonize the phenotypes between the cross-sectional and longitudinal treatment reports, we adopted the point-prevalent smoking checked at the 6-month or 12-month follow-up for these treatment studies, except for four studies in which Tashkin *et al.*^50^ reported the characteristics of the sustained quitters and continuing smokers at baseline. Stapleton *et al.*^52^ verified abstinence during weeks 3 and 4, Han *et al.*^49^ checked the smoking status at 4 weeks after bupropion administration and the smoking cessation rate of participants at 10 weeks as reported by Wilcox *et al.*^55^ were adopted.

### Data analysis

In consideration of the sources of heterogeneity, including study designs, ethnicity and participants' health conditions, stratified meta-analysis was performed first. Next, we pooled all the selected studies to implement a meta-analysis for the combined efforts. The significance of pooled OR is determined by a *Z*-statistical test, and exact *P*-values are presented throughout. All the pooled ORs were analyzed under both the fixed- and random-effects models with a 95% CI as described by DerSimonian and Laird.^58^ The hypothesis of between-study homogeneity was rejected if the *P-*value for the *Q*-statistical test was <0.05, and the *I*^2^ statistical test was used to determine the degree of variation across studies resulting from heterogeneity rather than chance. Heterogeneity among studies was assumed if the *I*^2^ value was >50%.^59^

Potential publication bias was evaluated graphically by a funnel plot, which assumes that studies with a larger sample size will be distributed near the average, whereas studies with a smaller sample will be equally distributed on both sides of the average. Deviation from this funnel-shaped distribution can indicate the presence of publication bias. The Egger regression test was applied to assess the significance of publication bias. Data were analyzed by the Comprehensive Meta-Analysis statistical software (version 2.0, Biostat, Englewood, NJ, USA).

## Results

### Description of the included studies

Through this stringent search strategy, we selected 23 eligible studies consisting of nine cross-sectional surveys^15, 17, 19, 20, 21, 22, 23, 46, 47^ and 14 longitudinal studies,^36, 38, 39, 40, 41, 43, 48, 49, 50, 51, 52, 53, 54, 55^ which were published between 1994 and 2014. A total of 11 151 participants were extracted from these papers, which included sample sizes from 76 to 2123 (Table 1). For the selected populations, 18 were predominantly of Caucasian origin, four concentrated on samples of East Asian ancestry and only one study, reported by Wu *et al.*,^21^ included persons of Mexican or African origin. The frequencies of A1/* genotypes showed large differences by ethnicity, being from 32.5 to 43.3% (mean=37.5%) in Caucasians and from 44.8 to 70.1% (mean=60.9%) in East Asians. In this meta-analysis, a total of 4411 participants were identified with A1/* genotypes with a frequency of 39.6%, which was in conformity with the frequencies in the Caucasian population. In addition, there was a wide distribution of cessation rates between 9.6 and 66.9%. The cessation rates of the cross-sectional studies and partial longitudinal studies, in which the smoking status was verified at end of treatment, ranged from 21.9 to 66.9%, whereas that of the longitudinal studies checked at 6- or 12-month follow-up were relatively lower (9.6–33.1%).

### Meta-analysis of cross-sectional studies

**Figure 1:**
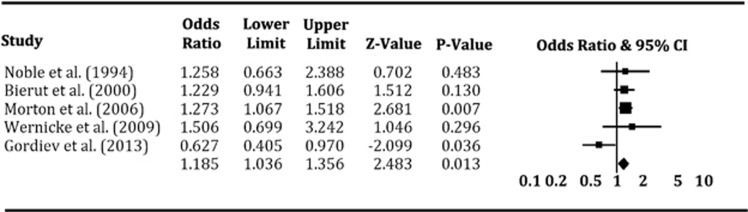
Forest plot of the meta-analysis results for the association between *Taq*1A A1/* genotypes and smoking cessation in cross-sectional studies based on predominantly Caucasian subjects. The *Z-*value and *P*-value of each eligible study are displayed by rows. The central vertical solid line stands for ORs that equal 1 for the null hypothesis. The OR and 95% CI of each study are represented by the square and horizontal bar, respectively. The pooled OR, which is represented by the diamond symbol underneath the forest plot was calculated under the fixed-effects model. CI, confidence interval; OR, odds ratio.

We initially conducted a meta-analysis to calculate the pooled effect of *Taq*1A A1/* genotypes on smoking cessation by combining 10 data sets extracted from nine cross-sectional studies in that the data set of the study by Wu *et al.*^21^ was divided into two parts according to ancestral origin. The pooled OR was 1.12 (*P*=0.07) with 95% CI ranging from 0.99 to 1.27 under the fixed-effects model and 1.03 (*P*=0.84) with 95% CI from 0.81 to 1.30 under the random-effects model (Table 2). Although results from the fixed-effects model suggested a trend to the genotypes of *Taq*1A A1/* correlating with a lower possibility of obtaining smoking cessation, there existed statistically significant heterogeneity among studies (*I*^2^=53.8% *P*=0.02). By taking into account the variation of ethnicity, we repeated the meta-analysis on the basis of predominant Caucasian ancestry, which revealed a significant association of *Taq*1A polymorphism with smoking cessation under the fixed-effects model (pooled OR 1.19; 95% CI 1.04–1.36; *P*=0.01; see Figure 1) and no significant association under the random-effects model with a moderate heterogeneity among studies (*I*^2^=57.0% *P*=0.05; Table 2).

Because only one study, reported by Gordiev *et al.*,^23^ recruited cancer patients, and this study also deviated from the funnel-shaped distribution (Supplementary Figure S5b), it is reasonable to postulate that the study of Gordiev *et al.* might contribute to the observed heterogeneity in the meta-analysis for the Caucasian population. After we excluded this study^23^ from the meta-analysis, the *P*-value of the *Q-*test increased from 0.05 to 0.97, and the *I*^2^ value was reduced from 57.4% to 0, indicating absence of between-study heterogeneity. There was a statistically significant association between *Taq*1A A1/* genotypes and smoking cessation for both fixed- and random-effects models (pooled OR 1.27; 95% CI 1.10–1.46; *P*=0.001 for both models). The forest plot is presented in Supplementary Figure S2.

### Meta-analysis of longitudinal studies

**Figure 2:**
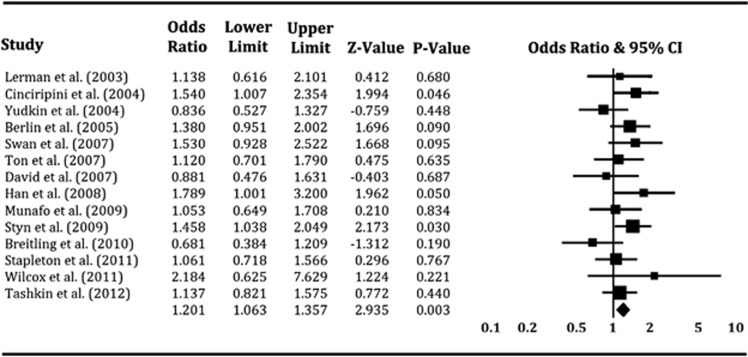
Forest plot of the meta-analysis results for the association between *Taq*1A A1/* genotypes and smoking cessation across all longitudinal studies. The *Z-*value and *P*-value of each eligible study are displayed by rows. The central vertical solid line stands for ORs that equal 1 for the null hypothesis. The OR and 95% CI of each study are represented by the square and horizontal bar, respectively. The pooled OR, which is represented by the diamond symbol underneath the forest plot was calculated under the fixed-effects model. CI, confidence interval; OR, odds ratio.

By pooling all the longitudinal studies, the OR in the fixed-effects model was 1.20 (*P*=0.003) with 95% CI of 1.06 and 1.36. In the random-effects model, the OR was 1.20 (*P*=0.007) with 95% CI of 1.05 and 1.37. There was no evidence of significant heterogeneity among studies (*I*^2^=11.0% *P*=0.33). For this part of the analysis, the results from the fixed-effects model seem to be more encouraging, indicating a statistically significant association of *Taq*1A A1/* genotypes with smoking cessation (Figure 2). When we performed the meta-analysis on samples with predominantly Caucasian ancestry, the results showed consistently that *Taq*1A A1/* genotypes were significantly associated with smoking cessation under both the fixed-effects (pooled OR 1.18; 95% CI 1.04–1.34; *P*=0.01) and the random-effects (pooled OR 1.17; 95% CI 1.03–1.34; *P*=0.01) models. The *P-*value of the *Q*-statistical test was 0.39, and the value of the *I*^2^ statistical test was 6.1%, indicating no evidence of between-study heterogeneity. These data are shown in Table 2, and the forest plot is presented in Supplementary Figure S3.

### Meta-analysis of combined studies

**Figure 3:**
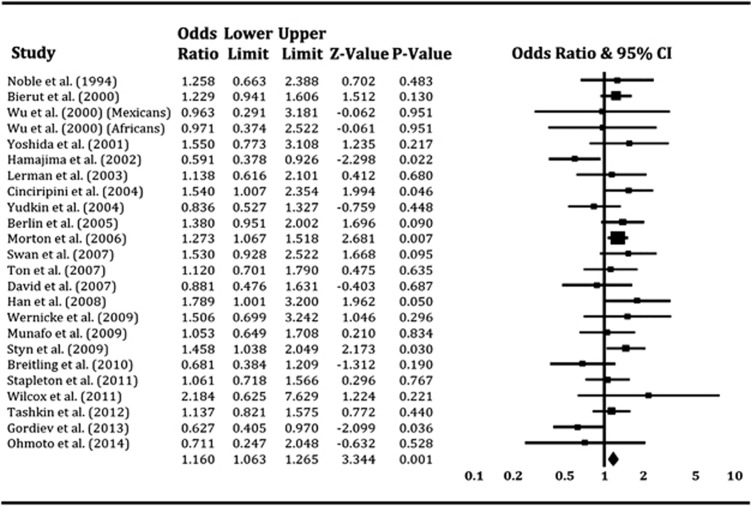
Forest plot of the meta-analysis results for the association between *Taq*1A A1/* genotypes and smoking cessation across all studies. The *Z-*value and *P*-value of each eligible study are displayed by rows. The central vertical solid line stands for ORs that equal 1 for the null hypothesis. The OR and 95% CI of each study are represented by the square and horizontal bar, respectively. The pooled OR, which is represented by the diamond symbol underneath the forest plot was calculated under the fixed-effects model. CI, confidence interval; OR, odds ratio.

Considering there were no significant differences in the pooled effects between the cross-sectional surveys and the longitudinal studies, we combined these studies to conduct a meta-analysis, as previously reported.^56^ For the overall population, the pooled OR was 1.16 (*P*=0.0008) with 95% CI of 1.06 and 1.27 under the fixed-effects model (Figure 3). There was evidence of marginally significant between-study heterogeneity (*I*^2^=33.9% *P*=0.055), but when repeating the meta-analysis under the random-effects model, we found that evidence for association remained significant (pooled OR 1.13; 95% CI 1.00–1.27; *P*=0.04).

Similarly, we carried out stratified meta-analyses for East Asian and Caucasian ancestry. Other ancestries, namely Mexicans or Africans, were excluded because there was only one study.^21^ With regard to East Asians, there was no evidence of significant association of *Taq*1A A1/* genotypes with smoking cessation (Table 2). When studies that contained subjects of primarily Caucasian origin were analyzed, the results indicated a statistically significant association between *Taq*1A A1/* genotypes and smoking cessation under the fixed-effects model (pooled OR 1.18; 95% CI 1.08–1.30; *P*=3 × 10^−4^). The forest plot is presented in Supplementary Figure S4. There was no evidence for significant heterogeneity among studies (*I*^2^=23.0% *P*=0.18). When we performed the meta-analysis under the random-effects model, the pooled OR was 1.16 (*P*=0.009) with 95% CI of 1.04 and 1.30. Again, after excluding the study of Gordiev *et al.*,^23^ the *I*^*2*^ value is reduced to 0, and the *P-*value of the *Q*-test increased to 0.63, indicating that the residual between-study heterogeneity has been eliminated. The results showed a more significant effect of *Taq*1A A1/* genotypes on smoking cessation under the fixed- and random-effects models (pooled OR 1.22; 95% CI 1.11–1.34; *P*=3.9 × 10^−5^ for both models; see Table 3).

### Sensitivity and accumulative analysis

**Figure 4:**
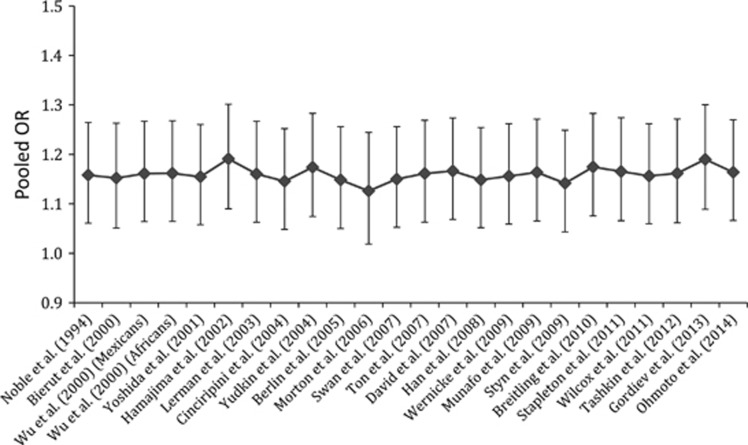
Plots of sensitivity analysis results for the meta-analyses across all studies. The *y* axis stands for the pooled OR, and the *x* axis for the individual study that was left out in sequence from the included studies. The diamond symbols represent the pooled OR, and the top and bottom horizontal bars mark the 95% CIs. CI, confidence interval; OR, odds ratio.

**Figure 5:**
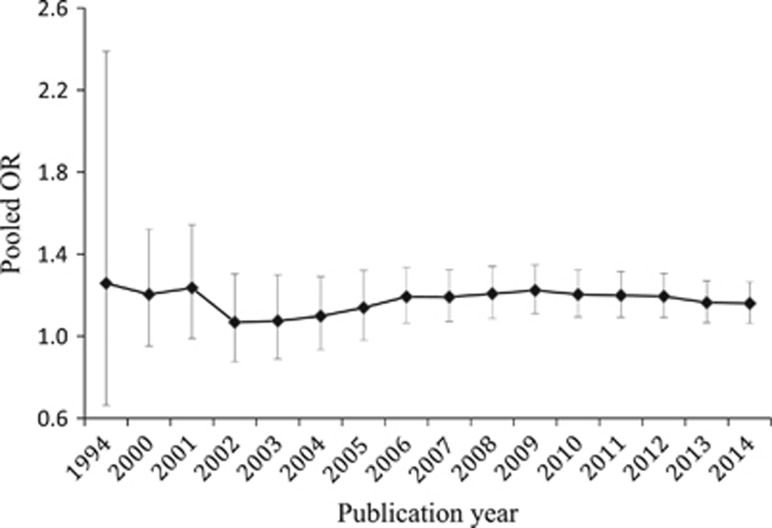
Plots of accumulative analysis results. The pooled effect size of the *Taq*1A polymorphism for the possibility of smoking cessation was plotted against the publication year across all studies. The *y* axis stands for the pooled OR and the *x* axis for the publication year relative to the pooled effect size. The diamond symbols indicate the pooled effect size, and each vertical line with horizontal bars labels the 95% CI. CI, confidence interval; OR, odds ratio.

We conducted a sensitivity analysis for the overall population of the combined studies under the fixed-effects model to examine whether the observed association of *Taq*1A polymorphism with smoking cessation was prominently influenced by leaving one individual study out at a time. As shown in Figure 4, the pooled ORs fluctuated faintly between 1.14 and 1.19, indicating the results of current meta-analysis were not significantly affected by any individual study. All the *P-*values for each examination of sensitivity were statistically significant (Supplementary Table S2). To determine whether the effect of the *Taq*1A polymorphism on smoking cessation changes with the publication year, we also carried out an accumulative analysis for overall population of combined studies under the fixed-effects model. The pooled OR decreased from 1.26 in 1994 to 1.19 in 2006 and achieved relatively steady values after that (Figure 5). The *P-*values and other detailed data are presented in Supplementary Table S3.

### Assessment of possible publication bias

We applied both statistical and graphic approaches to assess the potential publication bias that might exist in the stratified and combined meta-analyses. As displayed in Table 2, the *P*-values from all Egger regression tests were more than 0.05, suggesting there was no significant publication bias in our meta-analyses. Also, the funnel plots showed a consistent result with no evidence for possible bias. For longitudinal studies, the funnel plots were symmetrical, indicating there was no evidence of ascertainment bias (Supplementary Figures S6a and b). For cross-sectional and combined studies, there was one or two studies of several plots deviating from the funnel-shaped distribution (Supplementary Figures S5a, S5b, S7a and S7b). By considering the influence of ethnicity and subjects' health, we removed studies for a more reliable effect of *Taq*1A polymorphism on smoking cessation. As for the meta-analyses based on predominantly Caucasians without the data of the Gordiev study,^23^ the funnel plots achieved symmetry (Supplementary Figures S5c and S7c).

## Discussion

In this study, we observed an effect of *Taq*1A polymorphism on smoking cessation. For the cross-sectional studies, the meta-analysis showed a statistically significant association of *Taq*1A A1/* genotypes with smoking cessation in the Caucasian population, which is consistent with the results of a previous meta-analysis reported by Ohmoto *et al.*^22^ Thus, the nonsignificant association for the first meta-analysis reported by Munafo *et al.*^28^ was probably attributable to the insufficient number of studies with relatively small samples. In the longitudinal treatment studies, we extended previous work in the pharmacogenetic field of smoking cessation, as current meta-analysis provided evidence for the significant association, although Choi and Shin^44^ did not observed a significant association of *Taq*1A polymorphism with smoking cessation therapy in nine longitudinal treatment studies. This difference might have been attributable to the fact that only the treatment subjects were employed, which limited the number of samples. With respect to the meta-analysis of the combined studies, a much stronger association signal of *Taq*1A A1/* genotypes with smoking cessation was detected after excluding the underlying heterogeneity. On the basis of previous and current scientific evidence, we conclude that *DRD2 Taq*1A polymorphism is statistically significantly associated with smoking cessation (pooled OR 1.22; 95% CI 1.11–1.34; *P*=3.9 × 10^−5^ for both models), indicating that individuals with the homozygous *Taq*1A A2/A2 genotype are more likely to be successful in abstinence from cigarette smoking than those with A1/A1 or A1/A2 genotypes.

Dopamine receptors consist of two subfamilies with different functions: D1-like, including D1 and D5, and D2-like, including D2, D3 and D4. In particular, the dopamine D2 receptor (encoded by *DRD2*) is one of the important components of the dopaminergic reward pathway. Many studies have concentrated on determining whether variants in *DRD2* confer susceptibility to addictive disorders including smoking-related phenotypes and other psychiatric disorders. Multiple lines of evidence^10, 11, 12, 13, 14, 15, 16^ have demonstrated that numerous variants in *DRD2* are significantly associated with smoking behaviors. The polymorphism of rs1800497, which historically has been referred to as ‘*DRD2 Taq*1A', although it is located in *ANKK1*, was widely investigated in many genetic association studies^9, 15, 18, 19, 20, 21, 22, 23, 24, 25, 26, 27^ for smoking behaviors, including cessation. Furthermore, the *Taq*1A A1 allele has been associated with regulation of the functions of the dopamine D2 receptor by reducing the densities and binding affinity,^32, 33, 34, 35^ which may be one of the underlying molecular mechanisms of the association of the *Taq*1A A2/A2 genotype with the higher likelihood of abstaining from smoking. Nevertheless, there is a question remaining to be addressed: how a variant in *Taq*1A residing ~9.5 kb downstream from *DRD2* could influence the expression of *DRD2*. One speculative hypothesis is that the variant acts as a proxy marker in linkage disequilibrium (LD) with causative variant(s) within *DRD2*. Zhang *et al.*^60^ documented that *Taq*1A was in strong LD with rs2283265 and rs1076560 of *DRD2* (*D*′=0.86). Those two intronic single-nucleotide polymorphisms have been significantly associated with addictions,^15, 61, 62, 63, 64, 65, 66^ schizophrenia^67, 68, 69, 70, 71^ and reduced density of D2S relative to D2L and D2 receptors.^60^

Another possible mechanism is that the *Taq*1A polymorphism may exert its effort on the dopaminergic reward pathway by directly altering the function of ANKK1 itself. The product of *ANKK1*, which contains a single serine/threonine kinase domain and 11 ankyrin repeats and is a member of a protein family involved in signal transduction, was suggested to affect the dopaminergic circuitry through signal transduction or other cellular responses.^72, 73^ In addition, the function of many proteins could be modulated by phosphorylation of key amino-acid residues, which would cause a change of affinity of the protein for ligands or other aspects of activity. Thus, ANKK1 might act on the dopamine transporter to affect its activity.^74^ The *Taq*1A polymorphism was reported to alter a glutamate-to-lysine substitution at amino-acid residue 713 in the putative binding domain of ANKK1, indicating the variant may directly tailor the function of ANKK1 and indirectly regulate the activity of the dopamine transporter. Consistent with this idea, several family-based association studies have demonstrated that a significant association of *ANKK1* with nicotine addiction,^10, 11, 13^ as well as the association signal from variants in *ANKK1* appears to be stronger than that in *DRD2*.^9^ Besides, the *Taq*1A polymorphism may serve as a surrogate in LD with causative variant(s) in *ANKK1*. For example, Gelernter *et al.*^10^ reported that *Taq*1A is in complete LD with rs11604671 (D′=1.0) and in moderate LD with rs4938015 (D′=0.73), which are two functional variants in *ANKK1*. Given that the function of ANKK1 in the dopaminergic reward pathway is still unclear, more related functional studies are warranted. Meanwhile, as next-generation sequencing technologies become available, gene-based deep-sequencing analysis of the DRD2/ANKK1 gene cluster can be used to reveal more novel functional variants, which may be in strong LD with the *Taq*1A polymorphism, conferring susceptibility to smoking cessation.

To avoid the influence of underlying heterogeneity among studies, several stepwise stratified analyses were carried out in our meta-analyses. We first implemented two subgroup meta-analyses by two methods before combining the two types of studies. There was no evidence that the observed effect for the cross-sectional studies and the longitudinal studies were heterogeneous, providing persuasive evidence for the robustness of the finding of our meta-analysis of combined studies. In light of the differences in the frequencies of the A1/* genotype among different ethnic groups, we speculated that the influence of ethnicity might be attributable to the detected heterogeneity among studies. After excluding the subjects of East Asian and other non-Caucasian origins, the between-study heterogeneity was obviously reduced even though not erased. The results of the meta-analysis showed a more significant association in the Caucasian population. Further, we excluded the study of cancer patients^23^ and performed another meta-analysis. The residual heterogeneity among studies was eliminated, indicating that the findings of our meta-analysis for the Caucasian population were much more reliable. Unfortunately, the current study had an insufficient number of studies to incorporate East Asian subjects in the estimate of pooled effect. In addition, it is worth noting that there were several whole-genome-wide association studies^75^ where the *Taq*1A polymorphism was not revealed as a variant significantly associated with smoking cessation at the genome-wide significant level. To some extents, this is not surprising to us at all as so far only limited loci were identified by genome-wide association studies even in a study with a sample size of >140 000.^75^ Many factors might have contributed to this phenomenon; for example, inconsistent definition of smoking cessation phenotype and high heterogeneity among different studies, and very conservative threshold for significance with stringent Bonferroni correction.

Although a statistically significant association of *Taq*1A A1/* genotypes with smoking cessation was revealed, the results of our meta-analysis should be interpreted with caution in view of the following potential limitations. First, although reconciling the phenotypes of the chosen studies to the degree we could, the longitudinal treatment studies had different time frames for abstinence assessment, which might influence the observed effect of *Taq*1A polymorphism on smoking cessation. Furthermore, various medicines were used in these treatments for smoking abstinence interventions, which might also affect the detected association of the variant with smoking cessation, even though this study was concentrated on the main effect of *DRD2* genotypes on smoking cessation. Second, there were different sex ratios and mean ages of participants among these studies, which may be contributable to limitations, as suggested by many previous studies.^24, 31, 34, 48, 76^ Finally, because there are an insufficient number of studies on other polymorphisms, we could not examine the gene-by-gene interactions by which the pathogenesis of complex addictive disorders, including cessation, could be influenced. For example, Lerman *et al.*^38^ indicated that there was a significant statistical interaction of *SLC6A3* × *DRD2* with smoking cessation at the end of treatment, such that smokers with *SLC6A3* 9-repeat and *DRD2* A2A2 genotypes were more likely to abstain from cigarette smoking than were those who carry *SLC6A3* non-9-repeat and *DRD2* A2A2 genotypes. Further, Swan *et al.*^43^ consistently reported that subjects with both A1/* and non-9-repeat genotypes were less likely to be abstinent from smoking at 12-month follow-up. Recently, a meta-analysis^57^ by our group documented that a significant effect of *SLC6A3* 3′-UTR VNTR 9-repeat genotypes on smoking cessation in Caucasians, with an estimated effect of a 9-repeat allele being a 17% increase in the likelihood of abstinence. We thus postulate that *DRD2 Taq*1A A2/A2 and *SLC6A3* 9/* genotypes could contribute interactively to the process of quitting smoking.

In summary, the results of the current meta-analyses reveal a statistically significant association between the *DRD2 Taq*1A polymorphism and smoking cessation in a large Caucasian population. In comparison with smokers carrying one or more A1 alleles, smokers with homozygous A2 alleles are more likely to remain abstinent from cigarette smoking. Our results provide supportive evidence for further investigation of personalized medicine for smoking cessation according to individual genotypes. However, research on this problem remains in its infancy, so more well-designed genetic association, pharmacogenetic and molecular functional studies are warranted to reveal the function of *ANKK1* in the dopaminergic reward system and the molecular mechanism of *Taq*1A polymorphism for the etiology of smoking cessation; examine the association of other variants located in *DRD2*/*ANKK1*, which are in LD with the polymorphism of *Taq*1A, with cessation; identify more novel causative variants associated with cessation; and explore the underlying molecular mechanism of their function on tailoring intervention in nicotine dependence.

## Figures and Tables

**Table 1:** Characteristics of each study included in this meta-analysis (*N*=11 151 participants)

*Study name*	*Publication year*	*Study design*	*Sample size*	*% Caucasian*	*% Male*	*% Cessation (measurement method)*	% A1/* genotype frequency
Noble *et al.*^17^	1994	Cross-sectional survey	172	100	53.7	66.9 (self-report)	41.9
Bierut *et al.*^20^	2000	Cross-sectional survey	954	76	44.8	59.3 (self-report)	36.6
Wu *et al.*^21^	2000	Cross-sectional survey	59	0 (100% Mexicans)	78.0	40.7 (self-report)	74.6
Wu *et al.*^21^	2000	Cross-sectional survey	72	0 (100% Africans)	67.8	48.6 (self-report)	62.5
Yoshida *et al.*^46^	2001	Cross-sectional survey	134	0 (100% Asians)	46.7	42.5 (self-report)	44.8
Hamajima *et al.*^47^	2002	Cross-sectional survey	359	0 (100% Asians)	43.6	37.1 (self-report)	59.9
Lerman *et al.*^38^	2003	Prospective (treatment)	425	100	46.2	11.3 (cotinine verified)	42.4
Cinciripini *et al.*^53^	2004	Prospective (treatment)	134	91.8	49.3	15~20 (cotinine verified)	43.3
Yudkin *et al.*^36^	2004	Prospective (treatment)	752	99.0	40.8	10.1 (cotinine/CO verified)	41.2
Berlin *et al.*^40^	2005	Prospective (treatment)	595	85.0	48.0	10~20 (CO verified)	41.3
Morton *et al.*^15^	2006	Cross-sectional survey	2123	88.8	50.0	50.1 (self-report)	37.2
Swan *et al.*^43^	2007	Prospective (treatment)	323	100	37.5	33.1 (self-report)	35.3
Ton *et al.*^51^	2007	Prospective (treatment)	586	93	0 (100% female)	15.9 (cotinine verified)	35.5
David *et al.*^39^	2007	Prospective (treatment)	291	100	48.8	16.8 (cotinine verified)	44.3
Han *et al.*^49^	2008	Prospective (treatment)	225	0 (100% Asians)	100	40 (CO verified)	70.1
Wernicke *et al.*^19^	2009	Cross-sectional survey	129	100	50.4	37.2 (self-report)	34.9
Munafo *et al.*^41^	2009	Prospective (treatment)	804	100	48	10.0 (cotinine verified)	36.1
Styn *et al.*^48^	2009	Prospective (non-treatment)	881	90	48	24.9 (CO verified)	32.5
Breitling *et al.*^54^	2010	Prospective (treatment)	562	90.1	48	9.6 (cotinine verified)	32.7
Stapleton *et al.*^52^	2011	Prospective (treatment)	419	85.2	40.8	53.6 (CO verified)	41.3
Wilcox *et al.*^55^	2011	Prospective (treatment)	76	100	NA	19.7 (CO verified)	40.8
Tashkin *et al.*^50^	2012	Prospective (treatment)	621	>95	63.3	46.9 (cotinine and CO verified)	37.7
Gordiev *et al.*^23^	2013	Cross-sectional survey	359	100	65.6	43.7 (self-report)	35.4
Ohmoto *et al.*^22^	2014	Cross-sectional survey	96	0 (100% Asians)	91.7	21.9 (self-report)	65.6

Abbreviations: CO, carbon monoxide; NA, not applicable.

**Table 2:** Results from the meta-analysis for smoking cessation stratified by different data sets

*Data set of meta-analysis (A1A1+A1A2* vs *A2A2)*	*Cohorts (*n*)*	*Sample size (*N*)*	*Estimate of heterogeneity*		*Fixed-effects model*			*Random-effects model*			B^a^
			I^*2*^*(%)*	P *(*Q*)*	*Pooled OR*	*95% CI*	P *(*Z*)*	*Pooled OR*	*95% CI*	P *(*Z*)*	P *(*B*)*
Cross-sectional data set^b^	10	4457	53.8	0.02	1.12	0.99–1.27	0.07	1.03	0.81–1.30	0.84	0.35
Cross-sectional data set^c^	5	3737	57.0	0.05	1.19	1.04–1.36	0.01	1.12	0.87–1.45	0.38	0.62
Cross-sectional data set^d^	4	3378	0	0.97	1.27	1.10–1.46	0.001	1.27	1.10–1.46	0.001	0.50
Longitudinal data set^e^	14	6694	11.4	0.33	1.20	1.06–1.36	0.003	1.20	1.05–1.37	0.007	0.87
Longitudinal data set^f^	13	6469	6.1	0.39	1.18	1.04–1.34	0.01	1.18	1.03–1.34	0.01	0.62
Combined data set^g^	24	11 151	33.9	0.055	1.16	1.06–1.27	0.0008	1.13	1.00–1.27	0.04	0.40
Combined data set^h^	18	10 206	23.0	0.18	1.18	1.08–1.30	3 × 10^−4^	1.16	1.04–1.30	0.009	0.44
Combined data set^i^	17	9847	0.0	0.63	1.22	1.11–1.34	3.9 × 10^−5^	1.22	1.11–1.34	3.9 × 10^−5^	0.53
Combined data set^j^	4	945	72.8	0.01	0.97	0.72–1.32	0.86	1.05	0.56–1.96	0.88	0.68

Abbreviations: CI, confidence interval; OR, odds ratio.

a Egger regression test for publication bias.

b Analysis of cross-sectional data extracted from all the cross-sectional surveys.

c Analysis of cross-sectional data based on Caucasian population.

d Analysis of cross-sectional data based on Caucasian population without the data of the Gordiev study.

e Analysis of longitudinal data extracted from all the longitudinal studies.

f Analysis of longitudinal data extracted based on Caucasian population.

g Analysis of combined data extracted from both the cross-sectional surveys and the longitudinal studies.

h Analysis of combined data based on Caucasian population.

i Analysis of combined data based on Caucasian population without the data of the Gordiev study.

j Analysis of combined data based on Asian population.

**Table 3:** Results of meta-analysis for the association between *Taq*1A A1/* genotypes and smoking cessation across combined studies based on predominantly Caucasian origin with the Gordiev study

*Study name*	*Sample size*	*% Weight*	*Odds ratio*	*95% Confidence interval*	*Z-test*	P*-value*
Noble *et al.*^17^	172	2.13	1.26	0.66–2.39	0.70	0.483
Bierut *et al.*^20^	954	12.26	1.23	0.94–1.61	1.51	0.130
Lerman *et al.*^38^	425	2.33	1.14	0.62–2.10	0.41	0.680
Cinciripini *et al.*^53^	134	4.86	1.54	1.01–2.35	1.99	0.046
Yudkin *et al.*^18, 36, 37^	752	4.11	0.84	0.53–1.33	−0.76	0.448
Berlin *et al.*^40^	595	6.32	1.38	0.95–2.00	1.70	0.090
Morton *et al.*^15^	2123	28.16	1.27	1.07–1.52	2.68	0.007
Swan *et al.*^43^	323	3.51	1.53	0.93–2.52	1.67	0.095
Ton *et al.*^43, 51^	586	3.99	1.12	0.70–1.79	0.47	0.635
David *et al.*^39^	291	2.31	0.88	0.48–1.63	−0.40	0.687
Wernicke *et al.*^19^	129	1.49	1.51	0.70–3.24	1.05	0.296
Munafo *et al.*^29, 41^	804	3.74	1.05	0.65–1.71	0.21	0.834
Styn *et al.*^48^	881	7.56	1.46	1.04–2.05	2.17	0.030
Breitling *et al.*^54^	562	2.66	0.68	0.38–1.21	−1.31	0.190
Stapleton *et al.*^52^	419	5.76	1.06	0.72–1.57	0.30	0.767
Wilcox *et al.*^55^	76	0.56	2.18	0.63–7.63	1.22	0.221
Tashkin *et al.*^50^	621	8.25	1.14	0.82–1.57	0.77	0.440
Pooled analysis	9847	100.00	1.22	1.10–1.33	4.11	3.9 × 10^−5^

Notes: test for heterogeneity: *χ*^2^=13.6, df=16 (*P*=0.63), *I*^2^=0%. Test for overall effect under both fixed-effects and random-effects model: *Z*=4.03 (*P*=3.9 × 10^−5^).
