# Supplementary material for: The significant association of Taq1A genotypes in DRD2/ANKK1 with smoking cessation in a large-scale meta-analysis of Caucasian populations
Source: Transl Psychiatry. 2015 Dec 1;5(12):e686–. doi: 10.1038/tp.2015.176 (PMC5068580; doi:10.1038/tp.2015.176)
Supplement: Supplementary Tables [file tp2015176x1.pdf]

Supplementary Table S1: Detailed sample size for each *Taq*1A genotype and other related characteristics reported in original studies.

Study Name	Sample Origin	Language of Study	Current Smoker		Former Smoker		Hardy-Weinberg Equilibrium (HWE)
			A1/* genotype	A2/A2 genotype	A1/* genotype	A2/A2 genotype	
Noble et al. (1994)	USA	English	26	31	46	69	0.428
Bierut et al. (2000)	USA	English	153	235	196	370	——
Wu et al. (2000) (Mexicans)	USA	English	26	9	18	6	0.119
Wu et al. (2000) (Africans)	USA	English	23	14	22	13	0.382
Yoshida et al.(2001)	Japan	English	38	39	22	35	0.757
Hamajima et al.(2002)	Japan	English	125	101	90	43	0.947
Lerman et al. (2003)	USA	English	161	216	19	29	>0.05
Cinciripini et al. (2004)	USA	English	NA	NA	NA	NA	0.279
Yudkin et al. (2004)	UK	English	273	397	37	45	0.43
Berlin et al. (2005)	USA	English	NA	NA	NA	NA	0.904
Morton et al. (2006)	USA	English	416	623	373	711	0.413
Swan et al. (2007)	USA	English	83	133	31	76	0.37
Ton et al. (2007)	USA	English	177	316	31	62	>0.05
David et al. (2007)	USA	English	106	136	23	26	>0.46
Han et al. (2008)	South	English	102	33	57	33	0.01

	Korea						
Wernicke et al. (2009)	Germany	English	31	50	14	34	>0.05
Munafo et al. (2009)	UK	English	262	462	28	52	0.6
Styn et al. (2009)	USA	English	228	434	58	161	<0.01
Breitling et al. (2010)	Germany	English	162	346	22	32	0.016
Stapleton et al. (2011)	UK	English	82	113	91	133	0.15
Wilcox et al. (2011)	USA	English	27	34	4	11	——
Tashkin et al. (2012)	USA	English	129	201	105	186	——
Gordiev et al. (2013)	Russia	English	62	140	65	92	0.16
Ohmoto et al. (2014)	Japan	English	48	27	15	6	0.89

Supplementary Table S2: Detailed information of sensitivity analysis for the meta-analyses across all the combined studies.

Removed Study Name	Odds Ratio (OR)	Lower Limit	Upper Limit	Z-Value	P-Value
Noble et al. (1994)	1.16	1.06	1.26	3.28	0.0010
Bierut et al. (2000)	1.15	1.05	1.26	3.02	0.0026
Wu et al. (2000) (Mexicans)	1.16	1.06	1.27	3.36	0.0008
Wu et al. (2000) (Africans)	1.16	1.06	1.27	3.36	0.0008
Yoshida et al. (2001)	1.15	1.06	1.26	3.21	0.0013
Hamajima et al. (2002)	1.19	1.09	1.30	3.86	0.0001
Lerman et al. (2003)	1.16	1.06	1.27	3.32	0.0009
Cinciripini et al. (2004)	1.15	1.05	1.25	3.00	0.0027
Yudkin et al. (2004)	1.17	1.07	1.28	3.55	0.0004
Berlin et al. (2005)	1.15	1.05	1.26	3.03	0.0024
Morton et al. (2006)	1.13	1.02	1.24	2.32	0.0201
Swan et al. (2007)	1.15	1.05	1.26	3.10	0.0019
Ton et al. (2007)	1.16	1.06	1.27	3.31	0.0009
David et al. (2007)	1.17	1.07	1.27	3.44	0.0006
Han et al. (2008)	1.15	1.05	1.25	3.08	0.0020
Wernicke et al. (2009)	1.16	1.06	1.26	3.25	0.0012
Munafo et al. (2009)	1.16	1.07	1.27	3.36	0.0008
Styn et al. (2009)	1.14	1.04	1.25	2.88	0.0039
Breitling et al. (2010)	1.17	1.08	1.28	3.58	0.0003
Stapleton et al. (2011)	1.17	1.07	1.27	3.36	0.0008
Wilcox et al. (2011)	1.16	1.06	1.26	3.27	0.0011
Tashkin et al. (2012)	1.16	1.06	1.27	3.26	0.0011
Gordiev et al. (2013)	1.19	1.09	1.30	3.84	0.0001
Ohmoto et al. (2014)	1.16	1.07	1.27	3.41	0.0007

Supplementary Table S3: Detailed information of accumulative analysis across all the combined studies

Published Year	Odds Ratio (OR)	Lower Limit	Upper Limit	Z-Value	P-Value
1994	1.26	0.66	2.39	0.70	0.4827
2000	1.20	0.95	1.52	1.55	0.1200
2001	1.24	0.99	1.54	1.87	0.0618
2002	1.07	0.88	1.30	0.65	0.5136
2003	1.07	0.89	1.30	0.75	0.4541
2004	1.10	0.93	1.29	1.13	0.2566
2005	1.14	0.98	1.32	1.72	0.0860
2006	1.19	1.06	1.34	3.04	0.0024
2007	1.19	1.07	1.32	3.23	0.0012
2008	1.21	1.09	1.34	3.53	0.0004
2009	1.22	1.11	1.35	4.08	0.0000
2010	1.20	1.09	1.32	3.80	0.0001
2011	1.20	1.09	1.32	3.84	0.0001
2012	1.19	1.09	1.31	3.91	0.0001
2013	1.16	1.07	1.27	3.41	0.0007
2014	1.16	1.06	1.27	3.34	0.0008
