# Supplementary material for: The significant association of Taq1A genotypes in DRD2/ANKK1 with smoking cessation in a large-scale meta-analysis of Caucasian populations
Source: Transl Psychiatry. 2015 Dec 1;5(12):e686–. doi: 10.1038/tp.2015.176 (PMC5068580; doi:10.1038/tp.2015.176)
Supplement: Supplementary Figures [file tp2015176x2.pdf]

Supplementary Figure S1

Flow diagram of studies selection in meta-analysis

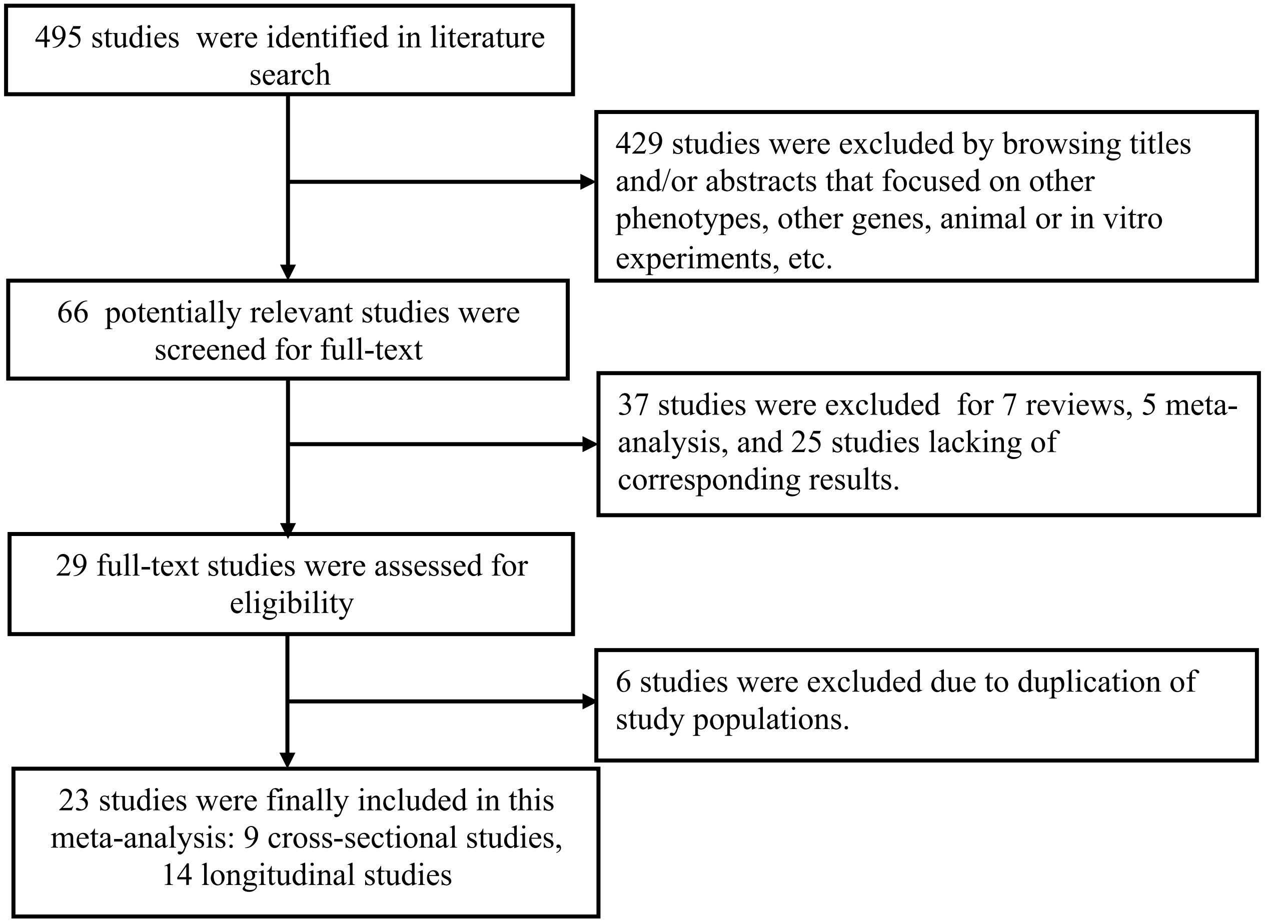

Figure S1

Supplementary Figure S2

Forest plot of the meta-analysis results for the association between *Taq*1A A1/* genotypes and smoking cessation across cross-sectional studies based on predominantly Caucasians without the Gordiev study. The Z-value and P-value of each eligible study are displayed by rows. The central vertical solid line stands for ORs that equal to 1 for the null hypothesis. The OR and 95% CI of each study are represented by the square and horizontal bar, respectively. The pooled OR, which is represented by the diamond symbol, underneath the forest plot was calculated under the fixed-effects model.

Study	Odds Ratio	Lower Limit	Upper Limit	Z-Value	P-Value
Noble et al. (1994)	1.258	0.663	2.388	0.702	0.483
Bierut et al. (2000)	1.229	0.941	1.606	1.512	0.130
Johnstone et al. (2004)	1.333	0.817	2.175	1.150	0.250
Morton et al. (2006)	1.273	1.067	1.518	2.681	0.007
Wernicke et al. (2009)	1.506	0.699	3.242	1.046	0.296
	1.272	1.111	1.456	3.478	0.001

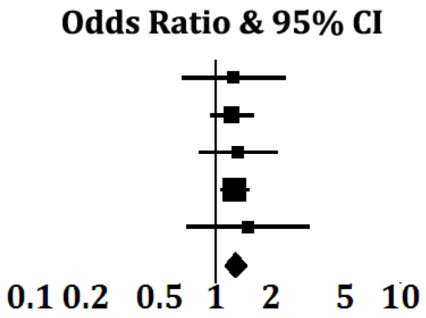

Figure S2

Supplementary Figure S3

Forest plot of the meta-analysis results for the association between *Taq*1A A1/* genotypes and smoking cessation across longitudinal studies based on predominant Caucasians. The Z-value and P-value of each eligible study are displayed by rows. The central vertical solid line stands for ORs that equal to 1 for the null hypothesis. The OR and 95% CI of each study are represented by the square and horizontal bar, respectively. The pooled OR, which is represented by the diamond symbol, underneath the forest plot was calculated under the fixed-effects model.

Study	Odds Ratio	Lower Limit	Upper Limit	Z-Value	P-Value
Lerman et al. (2003)	1.138	0.616	2.101	0.412	0.680
Cinciripini et al. (2004)	1.540	1.007	2.354	1.994	0.046
Yudkin et al. (2004)	0.836	0.527	1.327	-0.759	0.448
Berlin et al. (2005)	1.380	0.951	2.002	1.696	0.090
Swan et al. (2007)	1.530	0.928	2.522	1.668	0.095
Ton et al. (2007)	1.120	0.701	1.790	0.475	0.635
David et al. (2007)	0.881	0.476	1.631	-0.403	0.687
Munafo et al. (2009)	1.053	0.649	1.708	0.210	0.834
Styn et al. (2009)	1.458	1.038	2.049	2.173	0.030
Breitling et al. (2010)	0.681	0.384	1.209	-1.312	0.190
Stapleton et al. (2011)	1.061	0.718	1.566	0.296	0.767
Wilcox et al. (2011)	2.184	0.625	7.629	1.224	0.221
Tashkin et al. (2012)	1.137	0.821	1.575	0.772	0.440
	1.179	1.040	1.336	2.580	0.010

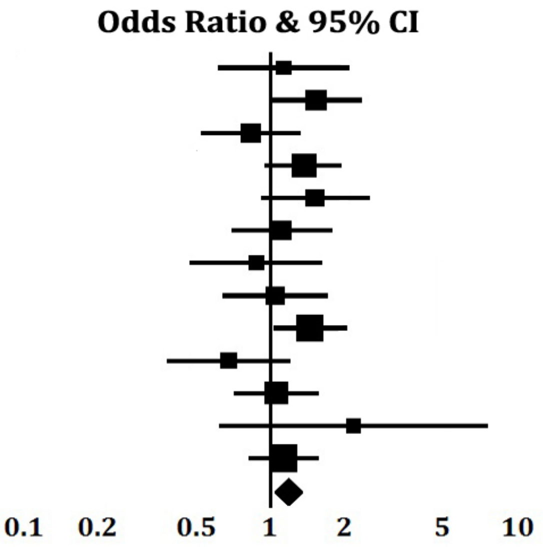

Figure S3

Supplementary Figure S4

Forest plot of the meta-analysis results for the association between *Taq*1A A1/* genotypes and smoking cessation across combined studies based on predominant Caucasians. The Z-value and P-value of each eligible study are displayed by rows. The central vertical solid line stands for ORs that equal to 1 for the null hypothesis. The OR and 95% CI of each study are represented by the square and horizontal bar, respectively. The pooled OR, which is represented by the diamond symbol, underneath the forest plot was calculated under the fixed-effects model.

Study	Odds Ratio	Lower Limit	Upper Limit	Z-Value	P-Value
Noble et al. (1994)	1.2581	0.6627	2.3882	0.7020	0.4827
Bierut et al. (2000)	1.2290	0.9408	1.6056	1.5124	0.1304
Lerman et al. (2003)	1.1377	0.6160	2.1010	0.4121	0.6803
Cinciripini et al. (2004)	1.5400	1.0075	2.3541	1.9943	0.0461
Yudkin et al. (2004)	0.8363	0.5272	1.3267	-0.7591	0.4478
Berlin et al. (2005)	1.3800	0.9511	2.0023	1.6960	0.0899
Morton et al. (2006)	1.2728	1.0670	1.5183	2.6810	0.0073
Swan et al. (2007)	1.5300	0.9283	2.5215	1.6681	0.0953
Ton et al. (2007)	1.1203	0.7010	1.7902	0.4748	0.6349
David et al. (2007)	0.8811	0.4759	1.6310	-0.4030	0.6870
Wernicke et al. (2009)	1.5057	0.6993	3.2419	1.0460	0.2956
Munafo et al. (2009)	1.0532	0.6492	1.7085	0.2099	0.8337
Styn et al. (2009)	1.4583	1.0377	2.0494	2.1730	0.0298
Breitling et al. (2010)	0.6810	0.3836	1.2091	-1.3117	0.1896
Stapleton et al. (2011)	1.0606	0.7182	1.5661	0.2958	0.7674
Wilcox et al. (2011)	2.1838	0.6251	7.6295	1.2238	0.2210
Tashkin et al. (2012)	1.1369	0.8208	1.5747	0.7719	0.4402
Gordiev et al. (2013)	0.6268	0.4052	0.9695	-2.0989	0.0358
	1.1819	1.0786	1:2952	3.5802	0.0003

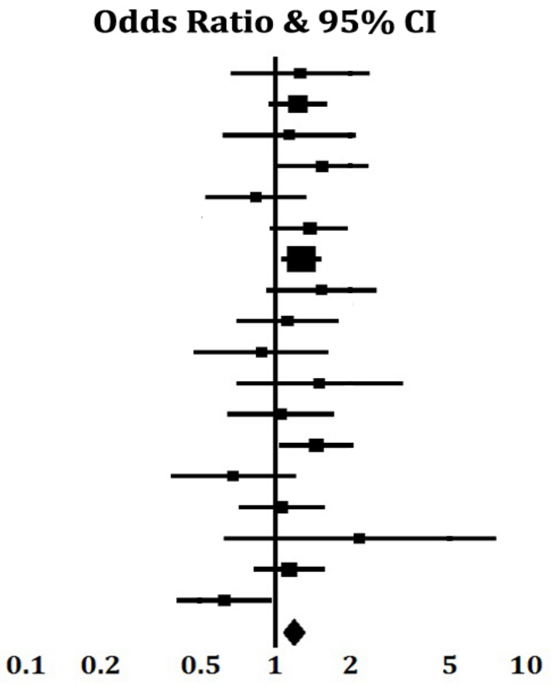

Figure S4

Supplementary Figure S5

Funnel plots of publication biases of cross-sectional studies; (S5a) all the cross-sectional studies, (S5b) cross-sectional studies based on predominant Caucasians, and (S5c) cross-sectional studies based on predominant Caucasians without the Gordiev study. Here, Y-axis stands for the standard error of the log odds ratio and X-axis stands for the log odds ratio. Each dot stands for an individual study.

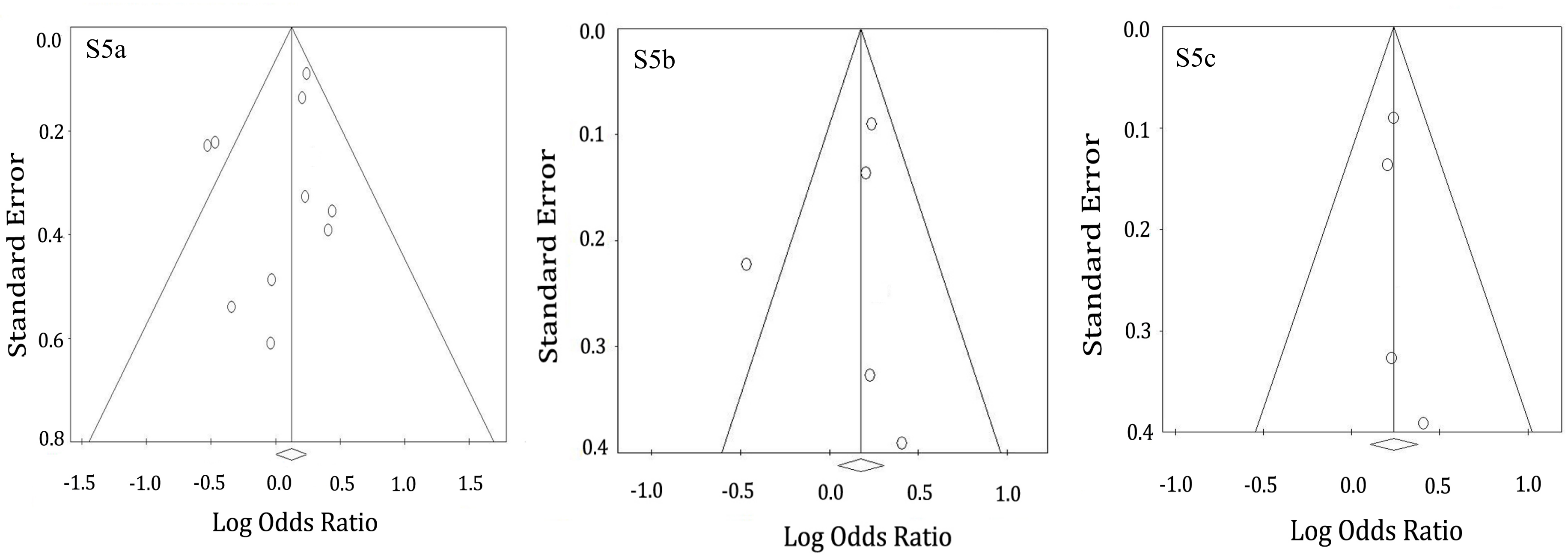

Figure S5

Supplementary Figure S6

Funnel plots of publication biases of longitudinal studies; (S6a) all the longitudinal studies, (S6b) longitudinal studies based on predominant Caucasians. Here, Y-axis stands for the standard error of the log odds ratio and X-axis stands for the log odds ratio. Each dot stands for an individual study.

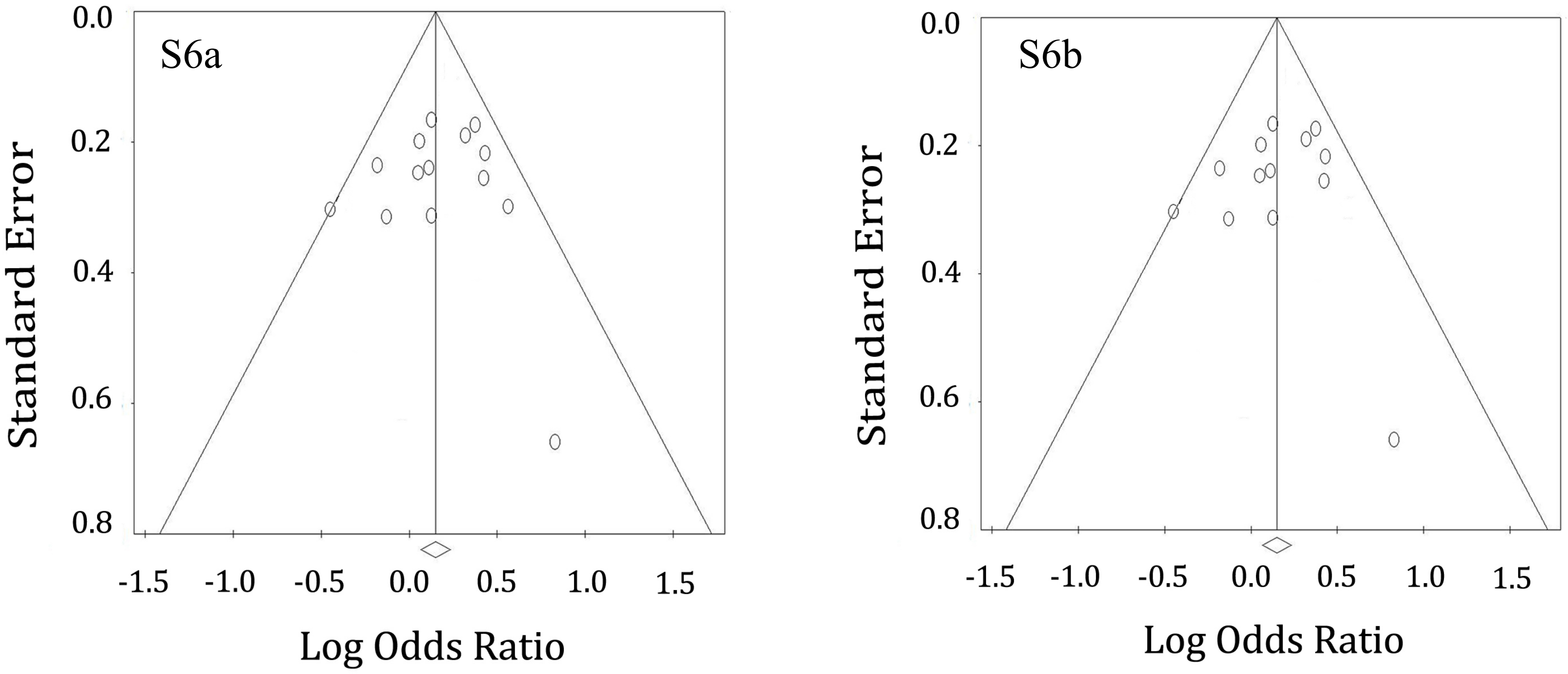

Figure S6

Supplementary Figure S7

Funnel plots of publication biases of combined studies; (S7a) all the combined studies, (S7b) combined studies based on predominant Caucasians, and (S7c) combined studies based on predominant Caucasians without the study of Gordiev et al. Here, Y-axis stands for the standard error of the log odds ratio and X-axis stands for the log odds ratio. Each dot stands for an individual study.

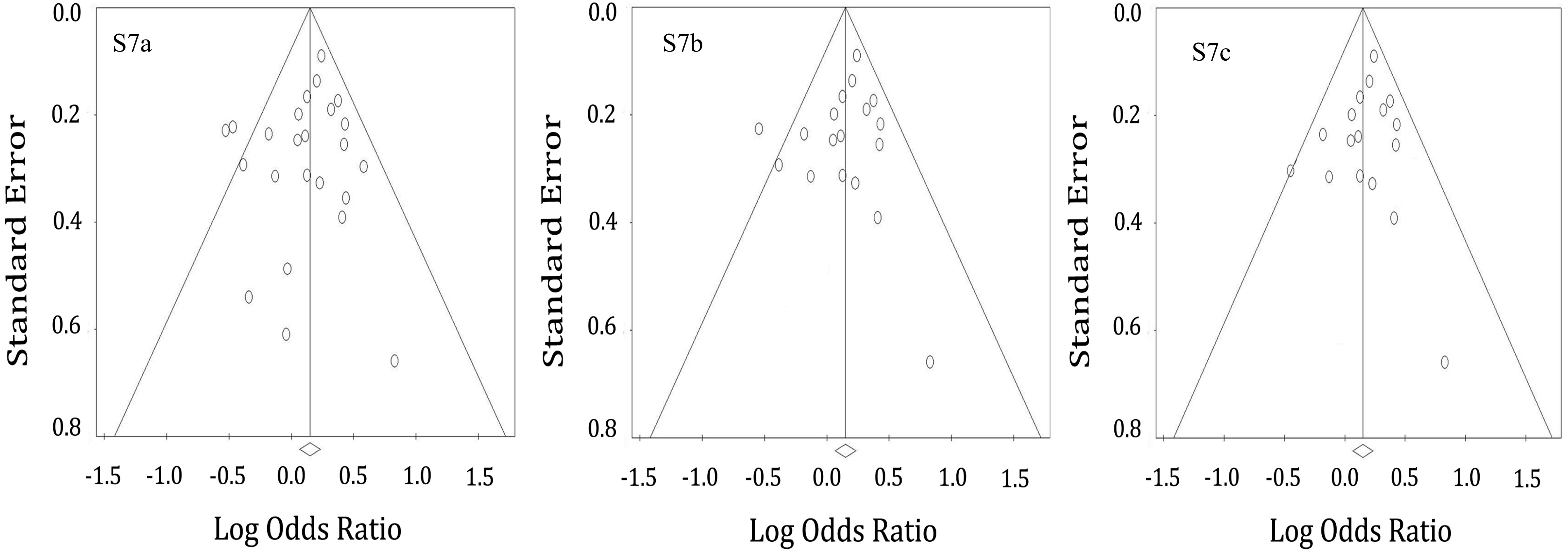

Figure S7
